# Imaging-cytometry revealed spatial heterogeneities of marker expression in undifferentiated human pluripotent stem cells

**DOI:** 10.1007/s11626-016-0084-3

**Published:** 2016-08-29

**Authors:** Mika Suga, Saoko Tachikawa, Daiki Tateyama, Kiyoshi Ohnuma, Miho K. Furue

**Affiliations:** 1 Laboratory of Stem Cell Cultures, National Institutes of Biomedical Innovation, Health and Nutrition, 7-6-8 Saito-Asagi, Osaka, Ibaraki 567-0085 Japan; 2 Department of Bioengineering, Nagaoka University of Technology, 1603-1 Kamitomioka, Nagaoka, Niigata 940-2188 Japan; 3 Research & Development Laboratory, NIPRO Corporation, 3023 Nojicho, Kusatsu, Shiga 525-0055 Japan

**Keywords:** Imaging, Cytometry, Human pluripotent stem cell, Quantitative characterization

## Abstract

Human pluripotent stem cells (hPSCs) provide a good model system for studying human development and are expected as a source for both cell-based medical and pharmaceutical research application. However, stable maintenance of undifferentiated hPSCs is yet challenging, and thus routine characterization is required. Flow-cytometry is one of the popular quantitative characterization tools for hPSCs, but it has drawback of spatial information loss of the cells in the culture. Here, we have applied a two-dimensional imaging cytometry that examines undifferentiated state of hPSCs to analyze localization and morphological information of immunopositive cells in the culture. The whole images of cells in a culture vessel were acquired and analyzed by an image analyzer, IN Cell Analyzer 2000, and determined staining intensity of the cells with their positional information. We have compared the expression of five hPSC-markers in four hPSC lines using the two-dimensional imaging cytometry and flow cytometry. The results showed that immunopositive ratios analyzed by the imaging cytometry had good correlation with those by the flow cytometry. Furthermore, the imaging cytometry revealed spatially heterogenic expression of hPSC-markers in undifferentiated hPSCs. Imaging cytometry is capable of reflecting minute aberrance without losing spatial and morphological information of the cells. It would be a powerful, useful, and time-efficient tool for characterizing hPSC colonies.

**Electronic supplementary material:**

The online version of this article (doi:10.1007/s11626-016-0084-3) contains supplementary material, which is available to authorized users.

## Introduction

Human pluripotent stem cells (hPSCs) including human embryonic stem cells (hESCs) (Thomson et al. 1998) and human induced pluripotent cells (hiPSCs) (Takahashi et al. 2007; Yu et al. 2007) are expected as a tool for studying human development as well as a source of both cell-based therapy and pharmaceutical research application for their ability to differentiate into almost all tissue cell types and to proliferate almost indefinitely (Thomson et al. 1998; Takahashi et al. 2007; Yu et al. 2007). Meanwhile, hPSCs are prone to spontaneous differentiation in a cell culture environment even if the same researcher cultures the same cell line under the same culture conditions (Thomson et al. 1998; Takahashi et al. 2007). Therefore, the cultured hPSCs should be routinely characterized for quality control of the cells (Furue 2009; Kusuda Furue et al. 2010; Hirata et al. 2011; The-International-Stem-Cell-Initiative 2011; Sheehy et al. 2014). The reproducibility of cell differentiation experiments strongly depends on the cell quality, which means the stemness of hPSCs. It was reported that the cardiac differentiation from hPSCs critically depended on the quality of the cells, which is reliant on the quality of both materials and the techniques employed (Lian et al. 2012). Thus, development of more valid quality evaluation method is an urgent issue for culturing hPSCs.

Flow cytometry is one of the most basic method for quantitative characterization of the quality of hPSCs. However, flow-cytometric analysis has two disadvantages for analyzing hPSCs. First, flow-cytometric analysis requires preparation of single cell suspension. In the case of hPSCs, it is difficult to dissociate tightly packed colonies, which is characteristic of undifferentiated hPSCs, into single cells uniformly. Moreover, since dissociated single hPSCs tend to die easily (Amit et al. 2000; Watanabe et al. 2007), procedure of flow cytometry is tricky and labor-intensive. Another problem involved is the loss of spatial information of the cells. Conventionally, researchers routinely check and empirically determine the quality of cells based on the spatial information obtained by the phase contrast microscopic observation, such as confluency, extent of differentiation, and tightness of cells in colonies. However, results based on flow cytometry sometimes contradict that of microscopic observation.

Imaging cytometry technology is expected to overcome these disadvantages of flow-cytometric analysis of hPSCs. Recent developments in digital imaging technology have enabled acquiring an entire surface image of a culture vessel and processing the image into numerical data. As a result, imaging cytometer is becoming a powerful tool for quantitative characterization of the cells (Eliceiri et al. 2012; Chieco et al. 2013). Since hPSCs form characteristic flat and monolayer colonies, imaging cytometry that can retain spatial information with quantitative analysis (Warmflash et al. 2014) is suitable to analyze individual hPSC colonies. Thus, daily phase contrast microscopic observation could be interpreted in relation to the quantitative analysis using imaging cytometry. Moreover, imaging cytometry analysis does not require preparation of single cell suspension.

Here, we have applied a two-dimensional imaging cytometry system to characterize hPSCs. We confirmed that our image cytometry system could characterize mixed population of cells in undifferentiated and differentiated state of hPSCs that we defined as the “quasi-undifferentiated state.” The hPSCs cultured and fixed in remote laboratory were subjected for analysis by our system to further standardize the analytical procedures. Consequently, the antigen expression profiles analyzed by the two-dimensional imaging cytometry were comparable with those by the flow cytometry. Quantitatively analyzed spatial information of the cells will help us understand the instability of undifferentiated hPSCs and control the quality of hPSCs.

## Materials and Methods

### hPSC culture

The information of the cells used in this study is summarized in Supplementary Table S1. The hESC H9 (WA09) (Thomson et al. 1998) line used at the National Institutes of Biomedical Innovation, Health and Nutrition (NIBIOHN) was obtained from WISC Bank (WiCell Research Institute, Madison, WI). The hiPSC 201B7 (Takahashi et al. 2007) and 253G1 (Nakagawa et al. 2007) lines used at Nagaoka University of Technology (NUTech) were obtained from RIKEN BRC Cell Bank (Ibaraki, Japan) through the National Bio-Resource Project for MEXT, Japan. NUTech also used hiPSC Tic line (JCRB1331) (Nagata et al. 2009) which was obtained from the Japan Collection of Research Bioresources Cell Bank (Osaka, Japan). The cells were defrosted, cultured, and routinely maintained in human ESC expansion medium (KSR-based medium) on mitomycin C-treated mouse embryonic fibroblast (MEF) feeder cells as previously described (Amit et al. 2000). KSR-based medium consisted of DMEM/F12 medium (Life Technologies, Carlsbad, CA), supplemented with 20% (*v*/*v*) KSR (Life Technologies), 0.1 mM 2-mercaptoethanol (Sigma, St. Louis, MO), MEM non-essential amino acids (Life Technologies), and 5–10 ng/ml recombinant human basic FGF (Katayama Kagaku Kogyo, Osaka, Japan). H9 cells were passaged using 1 mg/ml dispase (Roche, Mannheim, Germany) in DMEM/F12 medium (Life Technologies) and a plastic scraper (Sumitomo Bakelite, Tokyo, Japan). 201B7, 253G1, and Tic cells were passaged using CTK medium (Suemori et al. 2006) consisting of 0.25% trypsin, 0.1% collagenase IV, 20% KSR, and 1 mM CaCl_2_ in phosphate-buffered saline (PBS).

Cell culture, flow-cytometric analysis, and imaging-cytometric analysis of the hESC H9 line were performed at NIBIOHN. Cell culture of the hiPSCs was performed at NUTech. The cells were seeded in two 6-well plates at the same passages. Flow-cytometric analysis of the hiPSCs in one of the 6-well plates was performed at NUTech, while the cells in the other 6-well plate were fixed with formaldehyde and sent via delivery service to NIBIOHN for imaging-cytometric analysis (Fig. S1).

The hESCs were used in NIBIOHN, in accordance with the Guidelines for Utilization of Human Embryonic Stem Cells of the Ministry of Education, Culture, Sports, Science and Technology of Japan after approval by the institutional ethical review board at NIBIOHN.

### Imaging cytometry

The imaging cytometry was performed using an image analyzer, IN Cell Analyzer 2000 (GE Healthcare, Buckinghamshire, UK), as described previously (Kinehara et al. 2013). Briefly, cells cultured in a 6-well plate were fixed with 4% formaldehyde and kept in PBS at 4°C. Before the hiPSCs were sent from NUTech to NIBIOHN, the cells were rinsed with PBS containing 0.5 mM CaCl_2_ and 0.5 mM MgCl_2_ (PBS^+/+^). Each well of 6-well plate was fully filled with PBS^+/+^ and tightly sealed with Parafilm (Pechiney Plastic Packaging, WI) to avoid air bubbles. After delivery from NUTech to NIBIOHN, the cells were confirmed to be neither peeled off from the plates nor broken by transportation. Subsequently, the cells were permeabilized and blocked with PBS containing 0.1% Triton X-100, 1% BSA, and then reacted with primary antibodies in the same solution. The primary and secondary antibodies used are listed in Supplementary Table S2. Hoechst 33342 (Life Technologies) was used for nuclei staining. The image analysis was achieved with the image analyzer and its analyzing software, Developer Toolbox v.1.9 software (GE Healthcare).

### Flow cytometry

Flow-cytometric analysis was performed as described previously (Ohnuma et al. 2014). Briefly, all cells were dissociated into single cell suspension using 0.02% (*w*/*v*) ethylenediaminetetraacetic acid tetrasodium salt dihydrate in PBS and fixed in 4% formaldehyde. Subsequently, the cells were permeabilized and blocked with PBS containing 0.1–0.2 % Triton X-100, 10 mg/ml BSA. Then, the cells were incubated with primary antibodies and the primary antibody binding was visualized with secondary antibodies. The phycoerythrin conjugated anti-feeder antibodies (130-096-094, Miltenyi Biotec, Bergisch Gladbach, Germany) were used to discriminate hPSCs from MEFs. Antibody information is listed in Supplementary Table S2. JSAN (Bay Bioscience, Hyogo, Japan) or FACSCanto (BD Biosciences, La Jolla, CA) was used for data acquisition.

## Results

### Phase-contrast microscopic observation

**Figure 1.**
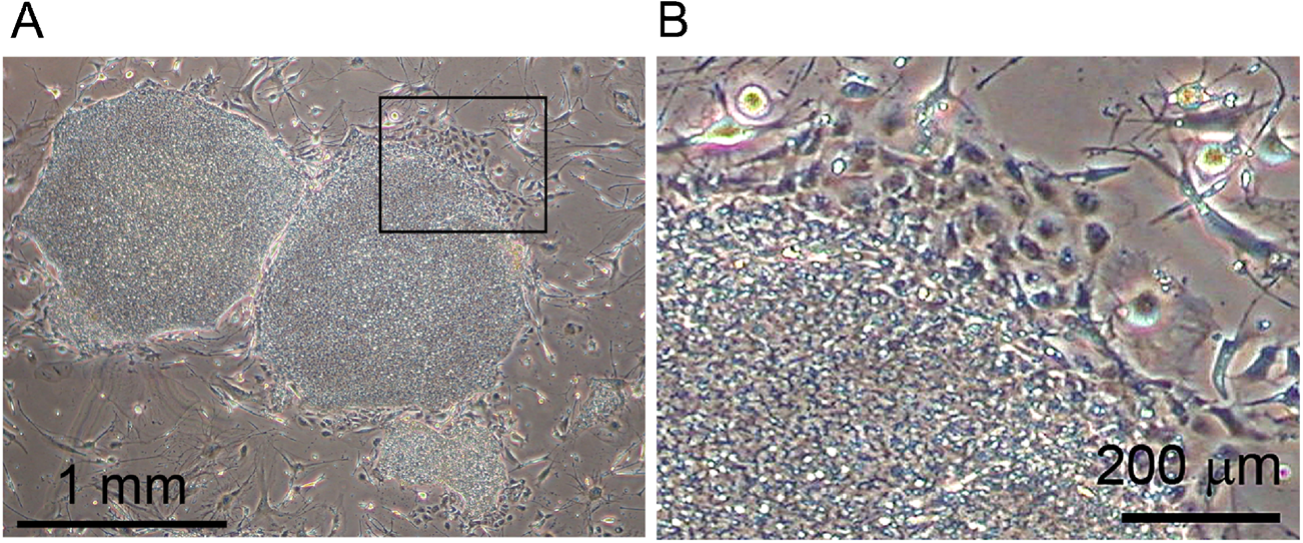
Morphology of phase contrast micrographs of hiPSCs 201B7. A representative phase contrast photograph of 201B7 cells. (*A*) Cells were cultured roughly in several passages and then seeded on a 6-well plate. (*B*) Magnified image of the boxed areas in (*A*). Note that the cells at the upper edge of the colony are spontaneously differentiated.

Human iPSCs, which were cultured roughly (i.e., with no particular efforts to force the cells to be in undifferentiated state such as picking up good colonies or removing differentiated cells) for several passages beforehand, were seeded on two 6-well plates. Phase contrast microscopic observation revealed that while most of the hiPSCs retained their undifferentiated state, which is characterized by tightly packed flat colonies of cells with large nuclei and scant cytoplasm (Thomson et al. 1998; Takahashi et al. 2007), small part of the cells underwent spontaneous differentiation, which is characterized by small black nuclei and extended cytoplasm (Thomson et al. 1998; Takahashi et al. 2007) (Fig. 1). We designated such composite cell population as “quasi-undifferentiated state” in this study.

### Imaging-cytometry analysis

The hiPSCs in one of the 6-well plates were delivered to NIBIOHN for imaging cytometry after fixation at NUTech (Fig. S1). H9 cells and the hiPSCs were double-immunostained with anti-OCT-3/4 and one of the following cell surface antibodies: stage-specific embryonic antigen (SSEA) 3, SSEA4, TRA-1-60, or SSEA1. OCT-3/4, SSEA3, SSEA4, and TRA-1-60 are undifferentiated stem cell markers (Thomson et al. 1998). SSEA-1 is an early-differentiated cell marker. Immuno-stained images of entire surface area of culture vessel were acquired to produce multi-panel tiling images (Fig. S4). The acquired tiled images were processed to recognize each cell area by the nuclear segmentation operation (Supplementary Table S3). Then, fluorescent intensity of Hoechst33342 (Nuclei, DAPI channel), Alexa Fluor 488 (anti-SSEA3, -SSEA4, -TRA-1-60, and -SSEA1 antibody, FITC channel), and Alexa Fluor 647 (anti-OCT 3/4 antibody, Cy5 channel) were measured at each of the processed nucleus area (Nuc area, Fig. S2 and Supplementary Table S3). Although many nuclei images were in contact with adjacent nuclei because of scant cytoplasm and tightly packed nature of hiPSCs, nuclei were clearly discriminated from each other by this procedure (Fig. S2).

### Marker expression profile by imaging-cytometric analysis

**Figure 2.**
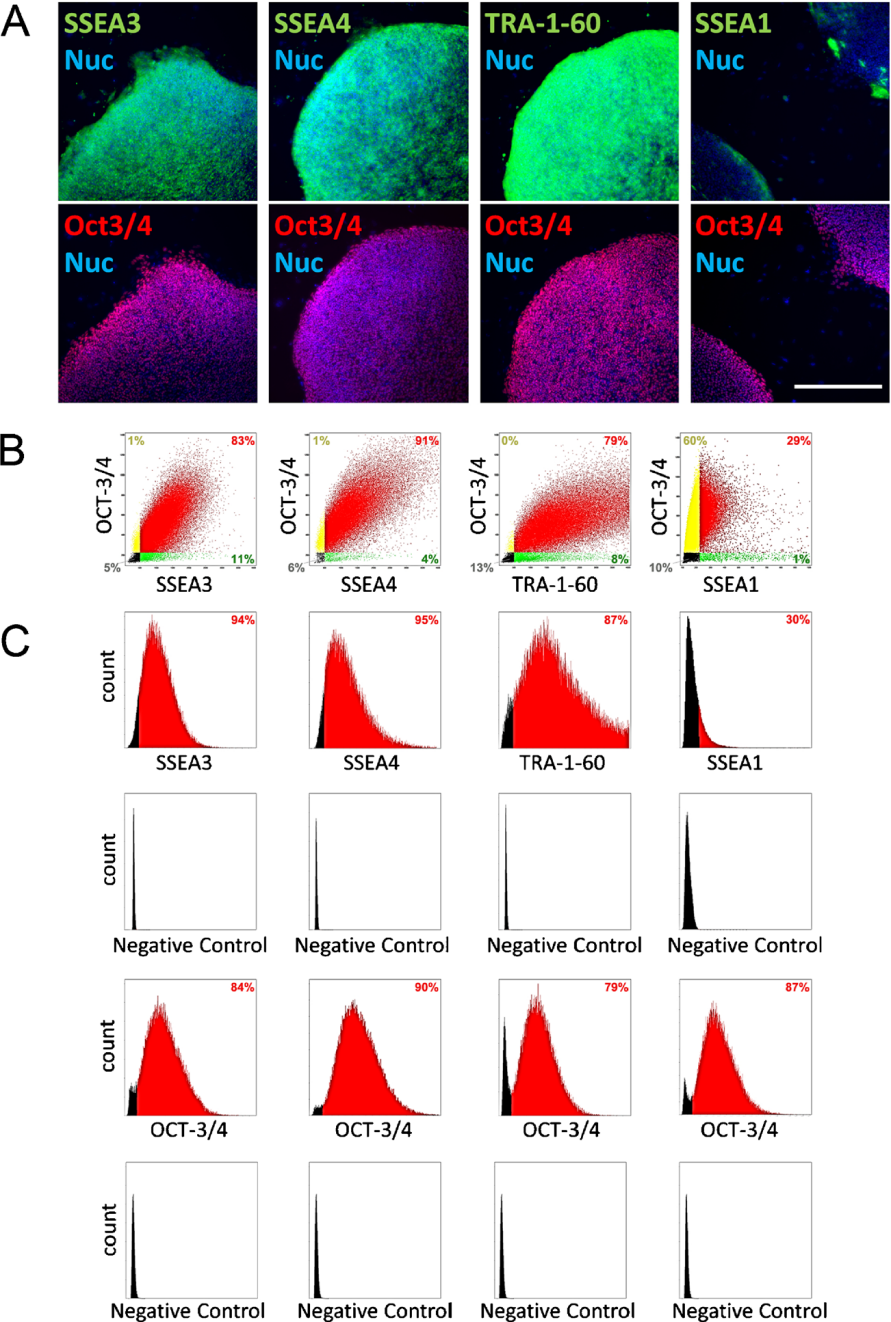
Imaging-cytometry analysis. Representatives of imaging-cytometric analysis of hiPSC 201B7. (*A*) The fluorescent images. Immunocytochemical images were taken by imaging cytometer. OCT-3/4 (*red*) and SSEA3, SSEA4, TRA-1-60, or SSEA1 (*green*) were double-stained. Nuclei were stained with Hoechst33342 (*blue*). *Scale bar*, 500 μm. (*B*) Two-dimensional plots of fluorescent intensity. The analyzed cells were plotted as double-positive (*red*), single-positive (*yellow and green*), or double-negative (*black*). *Data* are represented as percentage of expression. (*C*) Expression profiles in histogram. *Positive* is colored as *red*, and *negative* as *black*. Negative control samples were assayed without primary antibody.

As described previously, undifferentiated cells were stained well with OCT-3/4, SSEA3, SSEA4, and TRA-1-60, whereas differentiated cells that were delaminated from or on the edges of the undifferentiated colonies were positive for SSEA1 and negative for OCT-3/4 (Fig. 2 *A*). Fluorescence intensities of SSEA-3, SSEA-4, TRA-1-60, or SSEA-1 were plotted against that of OCT-3/4 bidimensionally (Figs. 2 *B* and S3), and expression profiles were obtained as histograms (Figs. 2 *C* and S3) in four hPSC lines, 201B7, 2531G1, Tic, and H9. A representative result of 201B7 cells (Fig. 2) showed that the percentage of positive cells for OCT-3/4, SSEA3, SSEA4, and TRA-1-60 were 85.2, 94.0, 95.0, and 87.0%, respectively, whereas SSEA1 positive cells was 30.2%, indicating that the cells were in “quasi-undifferentiated state.” The results of all experiments analyzed in this study are summarized in Supplementary Table S4.

### Strong correlation between imaging-cytometric analysis and flow-cytometric analysis

**Figure 3.**
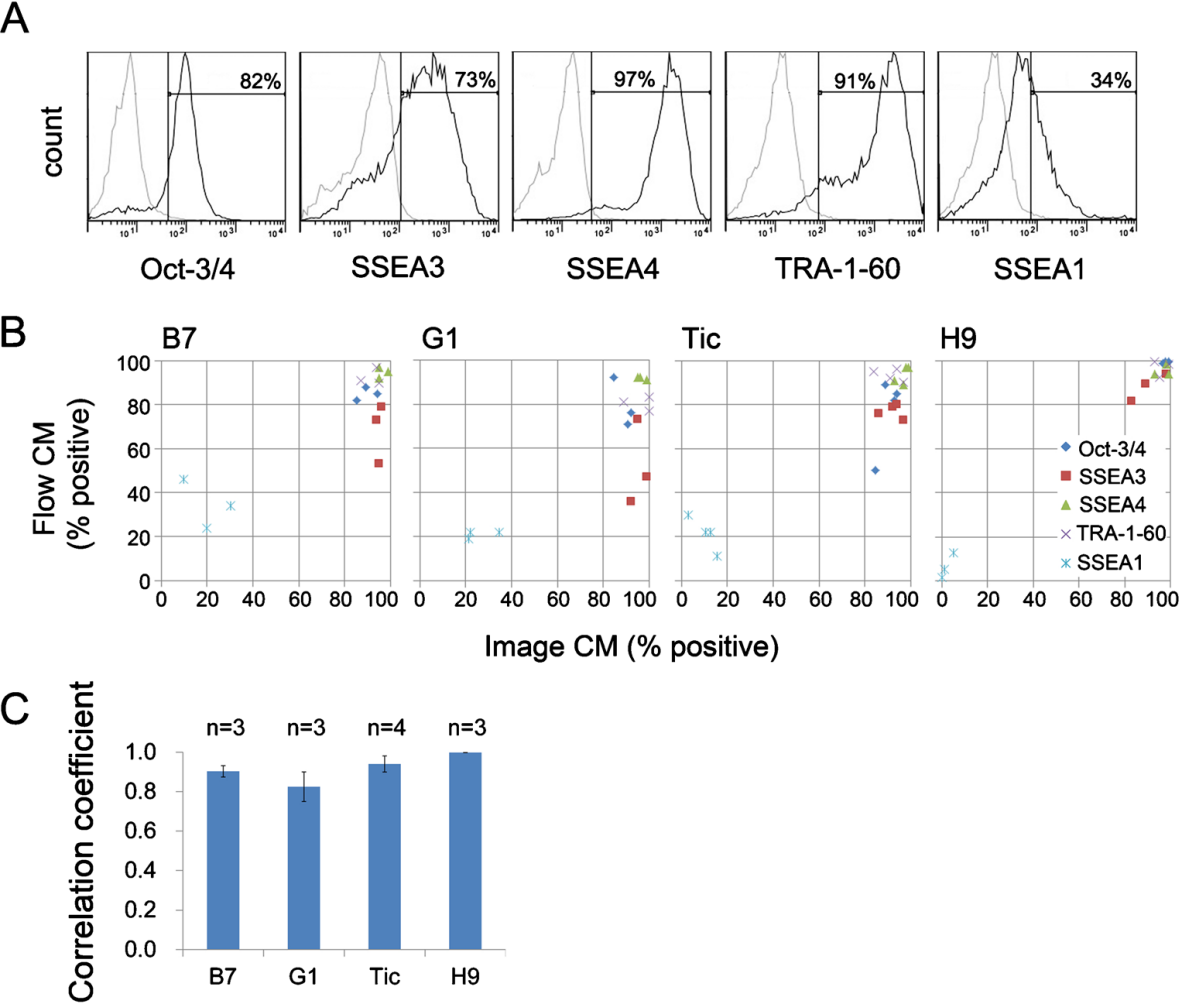
Correlation between imaging and flow cytometric analyses. (*A*) Representatives of flow-cytometry profiles of hiPSCs 201B7. *Dotted line*: negative control (without second antibody). *Solid line*: signal (with second antibody). *Horizontal lines* indicate gating areas for positive cells. (*B*) Percentage of immunopositive cells based on the imaging cytometry (abscissa) and flow cytometry (ordinate) of the sister cultured hiPSC 201B7 (B7), 253G1(G1) and Tic, and hESC H9. *CM* cytometry. (*C*) Pearson’s correlation coefficient between percentages of positive cells based on the imaging cytometry and flow cytometry of the four hPSC lines. The *numbers* on the column represent the number of independent experiments (mean ± se)

To determine whether antigen expression profiles analyzed by the imaging cytometry were adequate, the cells cultured in the other 6-well plate were analyzed in parallel with flow cytometry and the result was compared. The flow-cytometric analysis provided the antigen expression profile as histogram (Fig. 3 *A*). Although the original percentages of each marker expression measured by imaging and flow cytometry were different individually, result of all five markers as a whole exhibited strong correlation (Fig. 3 *B* and Table S4). The strong correlation was confirmed by calculating Pearson’s correlation coefficient (*r* = 0.95 ± 0.016, mean ± SE, *n* = 3: Fig. 3 *C*, 201B7). The mean value of Pearson’s correlation coefficient for all four hPSC lines was more than 0.7 (Fig. 3 *C*). Thus, imaging cytometry can quantitatively reflect results of “quasi-undifferentiated state” of hPSCs as with flow cytometry.

### Localization of stem cell markers in the hPSC colonies

One of the great advantages of imaging cytometry compared to flow cytometry is the conservation of spatial information of the cells. Because our imaging cytometry system keeps the link between the original fluorescent images and the cytometry profiles that give fluorescence intensity of each cell, it allows backtracking from profile to the image, showing exactly where the immuno-positive and immuno-negative cells for hESC markers are located in the original fluorescent image. Taking this advantage, we tried to elucidate where the immuno-positive/negative cells for OCT-3/4 with SSEA3, SSEA4, TRA-1-60, or SSEA1 located in the culture.

**Figure 4.**
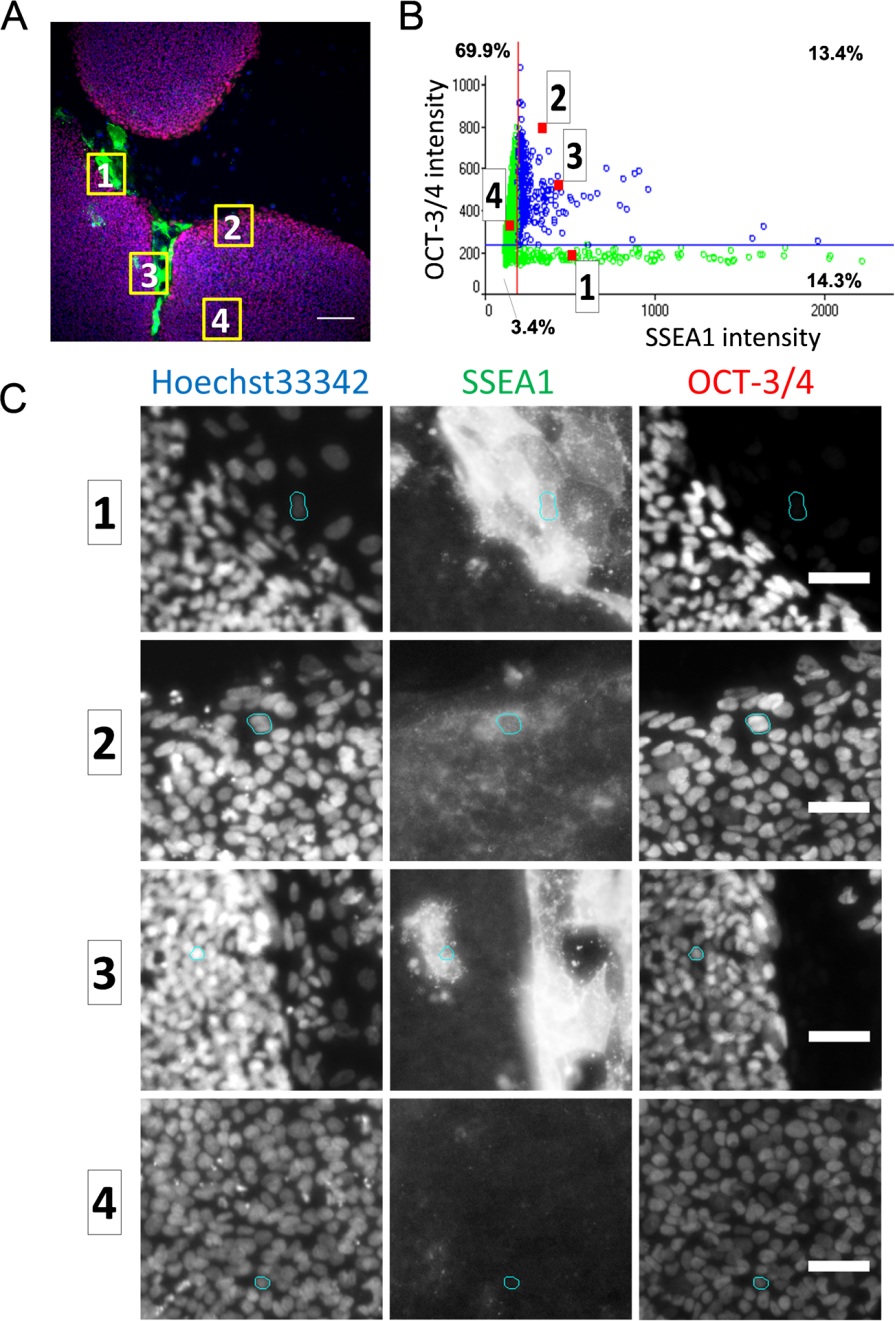

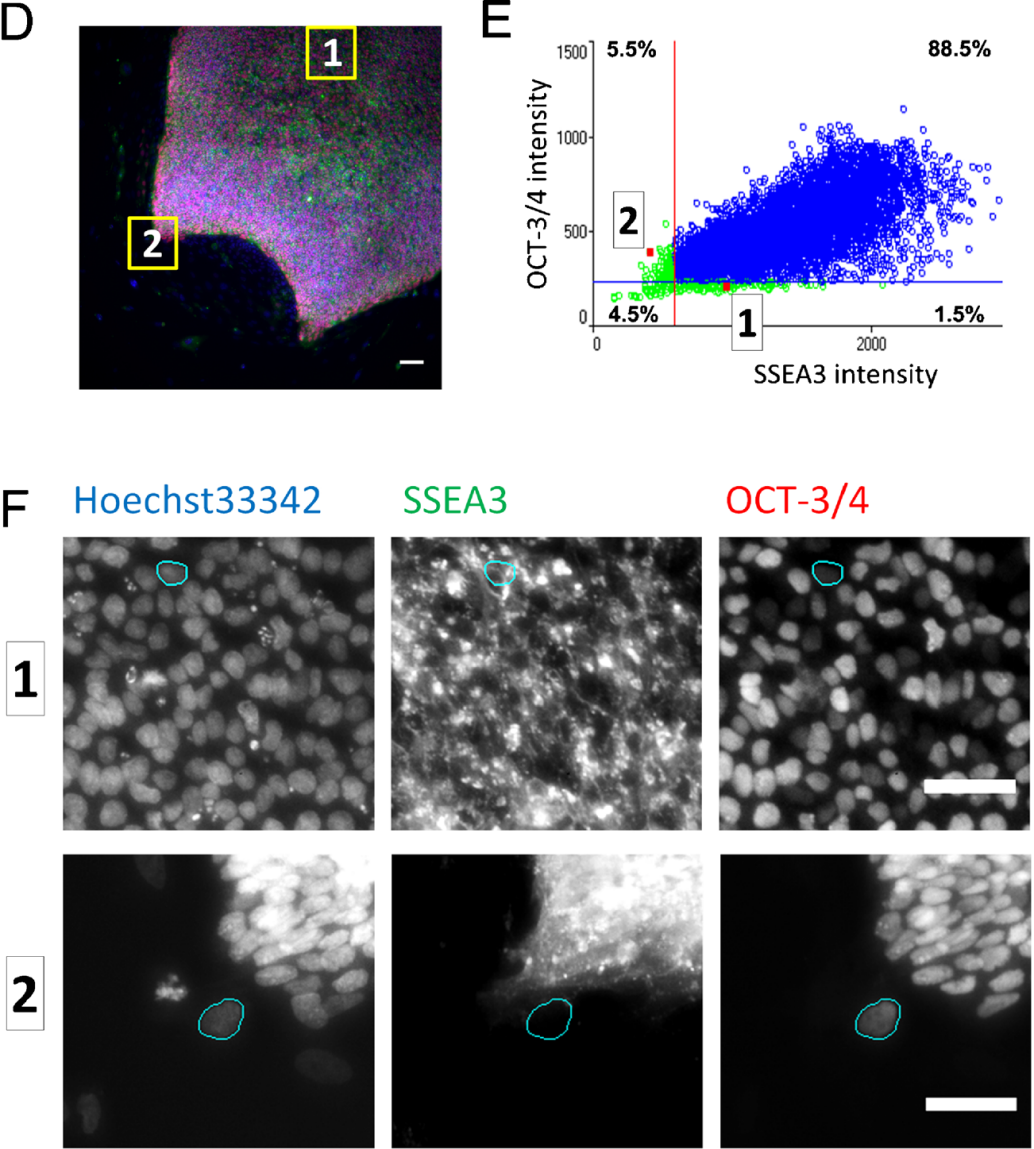
Localization of stem cell markers in hiPSC 201B7 colonies analyzed for expression profiles. Representative cells stained with Oct3/4 and SSEA1 (*A*–*C*) and with OCT3/4 and SSEA3 (*D*–*F*) were tracked back to the images from plots. (*A*, *D*) Merged image of immunostaining. A representative field of the images taken by imaging-cytometer is shown. Region containing each target cell is marked as *yellow rectangle*. *Scale bar*, 100 μm. (*B*, *E*) Analyzed fluorescent intensity profile of each cells in the field. *Red plots* represent target cells. (*C*, *F*) Fluorescent images stained with SSEA1, or SSEA3 with OCT-3/4 antibody and Hoechst33342. Nuclei region of each target cell is marked as *cyan*. (*C*) Representative plots for SSEA1 and OCT-3/4 were the following: *1*: SSEA1(+)/Oct-3/4(−) cell, *2* and *3*: SSEA1(+)/Oct-3/4(+) cell, *4*: SSEA1(−)/Oct-3/4(+) cell. (*F*) Representative plots for SSEA-3 and OCT-3/4 were the following: *1*: SSEA3(+)/Oct-3/4(−) cell; *2*: SSEA3(−)/Oct3/4(+) cell.

Bidimensional plots (Figs. 2 *B* and S3) and histogram (Figs. 2 *C* and S3) revealed the presence of single-positive cell population in the culture, although most cells were double-positive for OCT-3/4 and each of the undifferentiated cell surface markers, SSEA3, SSEA4, or TRA-1-60. Double-positive cells for OCT-3/4 and a differentiated cell surface marker, SSEA-1, were also present in the culture, though most cells were OCT-3/4-positive and SSEA1-negative. Tracking back to the original images from the plots in one of the field of view in 201B7 cell culture showed variable localization of marker expression. A SSEA1-single-positive cell indicated as “1” (Fig. 4 *B*) was localized at the peripheral of colony (Figs. 4 *A*, *C* and S4A). Double-positive cells for OCT-3/4 and SSEA1 indicated as “2” and “3” (Fig. 4 *B*) were localized in the colony (Figs. 4 *A*, C and S4A). An OCT-3/4-single-positive cell indicated as “4” (Fig. 4 *B*) was localized in the center of colony (Fig. 4 *A*, *C* and S4A). In another field of view, a SSEA-3 single-positive cell indicated as “1” (Fig. 4 *E*) was localized in the inner part of colony (Figs. 4 *D*, *F* and S4B) while an OCT-3/4-single-positive cell indicated as “2” (Fig. 4 *E*) was localized in the peripheral of colony (Figs. 4 *D*, *F* and S4B). These analyses indicated the heterogenic state of undifferentiated hPSCs in the culture. These results were consistent with the impression by observation under the phase-contrast microscope, suggesting that daily microscopic observation could be interpreted in relation to the quantitative analysis using imaging-cytometry.

## Discussion

In this study, we applied a two-dimensional imaging cytometry that can analyze the mixed population of undifferentiated and differentiated cells that we defined as the “quasi-undifferentiated state,” and its efficacy was confirmed by flow cytometry.

At first, we found that the detection sensitivity was different between imaging and flow cytometry. The results of image analysis depended highly on image processing parameters such as background subtraction, threshold of signal, and segmentation of cells. Moreover, the dynamic range of camera used in our imaging cytometry system was 4096 (12 bit camera), while the dynamic range of flow cytometry, which use photomultiplier and logarithm amplifier for detecting fluorescent signal, was 10,000 (display digit is 4). The narrowness of dynamic range in imaging cytometry system may distort the data profile at the lowest or highest part of the graph. However, although the percentage of each marker analyzed by the imaging cytometry was not exactly the same as that by the flow cytometry, the expression pattern of all five markers examined in this study showed strong correlation, indicating imaging cytometry can be used as flow cytometry for diagnosing hPSCs.

To determine expression profiles of undifferentiated markers of hPSCs, it is ideal to align detection sensitivity between experiments. The same holds for research collaboration, but difference between imaging cytometry and flow cytometry as well as models of analyzer employed affect detection sensitivity. In this study, this issue was avoided by delivering fixed hPSCs from NUTech to NIBIOHN where imaging cytometric analysis was performed. Thus, imaging cytometry can be used to analyze antigen expression profiles of hPSCs among collaborators.

Using imaging cytometry system, we succeeded to trackback from cytometric profile data to original fluorescent image to know where the immune-positive/negative cells were located, demonstrating that imaging cytometry is suitable for detecting spatial information. The image analysis in this study revealed that the cells positive for SSEA3, SSEA4, or TRA-1-60 but negative for Oct3/4 were located in both the center and peripheral area of the colonies. It was reported that the spontaneous differentiation of hPSCs are frequently found at limited areas in the colony such as at the edge or the center but not scattered across a colony (Thomson et al. 1998; Kim et al. 2014). Warmflash et al. reported that in situ imaging analysis revealed that BMP4 triggers spatially organized differentiation in hPSC colonies (Warmflash et al. 2014). To analyze cell differentiation of hPSC colonies in details, they used conventional fluorescent microscope for acquiring high magnification images and original data analysis programs using a numerical computing software, MATLAB (MathWorks, Natick, MA). In essence, their analysis method and performance would be comparable to our method using IN Cell Analyzer 2000, although our image analysis places priority on time efficiency over detailed analysis. Their findings also demonstrate that imaging cytometry is valuable to analyze cell differentiation process of hPSCs, although flow cytometry has a high sensitivity for quantifying antigen expression profiles.

In the present study, fixed cells were employed to establish basic technology for quantitative analysis of hPSCs using imaging cytometry. Fixed cells were capable of analyzing both surface markers and transcription factors, enabling multilateral analysis. Also, utilization of fixed cells enabled comparison of variety of hPSCs cultured in different laboratories on the same platform by collecting the fixed cells to a single laboratory to be analyzed. At the same time, living cell analysis would be valuable for daily quality check of hPSCs. We previously reported live cell imaging tools including antigens for florescent-labeled lectin, mitochondrial membrane potential probes, and non-labeling analyzing methods (Tateno et al. 2013; Suga et al. 2015; Kumagai et al. 2016). However, quantitative living cell analysis using imaging cytometry is more challenging than fixed cells for the time being. As shown in Table 1, our current protocol requires nuclei staining. Even though some kinds of dyes are able to stain live nuclei, most of them are toxic for living hPSCs. We are planning to develop living cell analysis in the future.

**Table 1.** Comparison of flow and imaging cytometry

	Flow-cytometry	Imaging cytometry
Sample preparation	Cell dissociation is needed but nuclear staining is unnecessary	Cell dissociation is unnecessary but nuclear staining are needed
Data from image	Allows quantitative and accurate single-cell-based analysis Loses spatial information of the cells	Provides spatial information of the cells such as morphology and localization in a colony False cell discrimination may misread data
Cell sorting	Well established Living cells can be sorted at a rate of more than thousands of cells per second	Underdeveloped
Usability	Data acquisition and analysis protocols are simple and well-established	Requires special knowledge or experiences for proper operation for it is a new technology

## Conclusion

We have applied a two-dimensional imaging cytometry to analyze spatial heterogeneity in undifferentiated state of the monolayer colony of hPSCs. Imaging cytometry can be used for quantitative analysis in equal measure to flow cytometry. In situ analysis without dissociation of cells allows obtaining spatial information of the cells, which may be useful for detecting spatial heterogeneity of hPSCs. The advantages of imaging cytometry allow quantifying condition of hPSCs easily and flexibly. We envision that imaging cytometry will be a standard procedure for medical applications of hPSCs.

## Electronic supplementary material

ESM 1 (DOCX 19 kb)

Figure S1 (PDF 72 kb)

Figure S2 (PDF 112 kb)

Figure S3 (PDF 269 kb)

Figure S4 (PDF 291 kb)

Table S1 (PDF 41 kb)

Table S2 (PDF 51 kb)

Table S3 (PDF 58 kb)

Table S4 (PDF 36 kb)
