# Supplementary material for: Imaging-cytometry revealed spatial heterogeneities of marker expression in undifferentiated human pluripotent stem cells
Source: In Vitro Cell Dev Biol Anim. 2016 Aug 29;53(1):83–91. doi: 10.1007/s11626-016-0084-3 (PMC5258813; doi:10.1007/s11626-016-0084-3)
Supplement: Supplementary file 1 — (DOCX 19 kb) [file 11626_2016_84_MOESM1_ESM.docx]

**Imaging-cytometry revealed spatial heterogeneities of marker expression in undifferentiated human pluripotent stem cells**

Mika Suga, Saoko Tachikawa, Daiki Tateyama, Kiyoshi Ohnuma, Miho K Furue

## Supplementary Information

Supplementary Figure S1: Schematic view of flow- and imaging-cytometric analysis of hPSCs

Supplementary Figure S2: Representative results of nuclei detection by image analysis

Supplementary Figure S3: Imaging-cytometric analysis of the hPSCs

Supplementary Figure S4: Tiled images obtained by the image analyzer

Supplementary Table S1: Information of hPSC lines used in this study

Supplementary Table S2: Antibody information

Supplementary Table S3: Steps in nuclear segmentation

Supplementary Table S4: Percent positive cells analyzed by imaging- and flow-cytometry

**Supplementary Figure Legends**

## Supplementary Figure S1. Schematic view of flow- and imaging-cytometric analysis of hPSCs.

Cell culture, phase-contrast micrography, and flow-cytometric analysis of the hESCs and the hiPSCs were done at NIBIOHN and NUTech respectively. All imaging-cytometric analysis were done at NIBIOHN with the fixed hPSCs of duplicate culture plates. Fixed hiPSCs were sent from NUTech to NIBIOHN via delivery service.

## Supplementary Figure S2. Representative results of nuclei detection by image analysis.

(A) Original obtained image. (B) Central region of each nucleus is marked as red (resultant of Nuc_center operation). (C) Extracted nucleus region is marked as cyan (resultant of Nuc operation).

## Supplementary Figure S3. Imaging-cytometric analysis of the hPSCs.

Representatives of imaging-cytometric analyses of (A) 253G1, (B) Tic and (C) H9 cell lines are shown.

## Supplementary Figure S4. Tiled images obtained by the image analyzer.

81 fields (9x9 fields) of obtained images are tiled. (A) Immunofluorescent images of 201B7 cells stained with Hoechst33342, SSEA1 and Oct-3/4. (B) Immunofluorescent images of 201B7 cells stained with Hoechst33342, SSEA3, and Oct-3/4. The field marked as yellow in (A) and (B) corresponds to Fig.3A and Fig.3D respectively. One field, 0.15 x 0.15 mm.
