# Supplementary figures and images for: Imaging-cytometry revealed spatial heterogeneities of marker expression in undifferentiated human pluripotent stem cells
Source: In Vitro Cell Dev Biol Anim. 2016 Aug 29;53(1):83–91. doi: 10.1007/s11626-016-0084-3 (PMC5258813; doi:10.1007/s11626-016-0084-3)

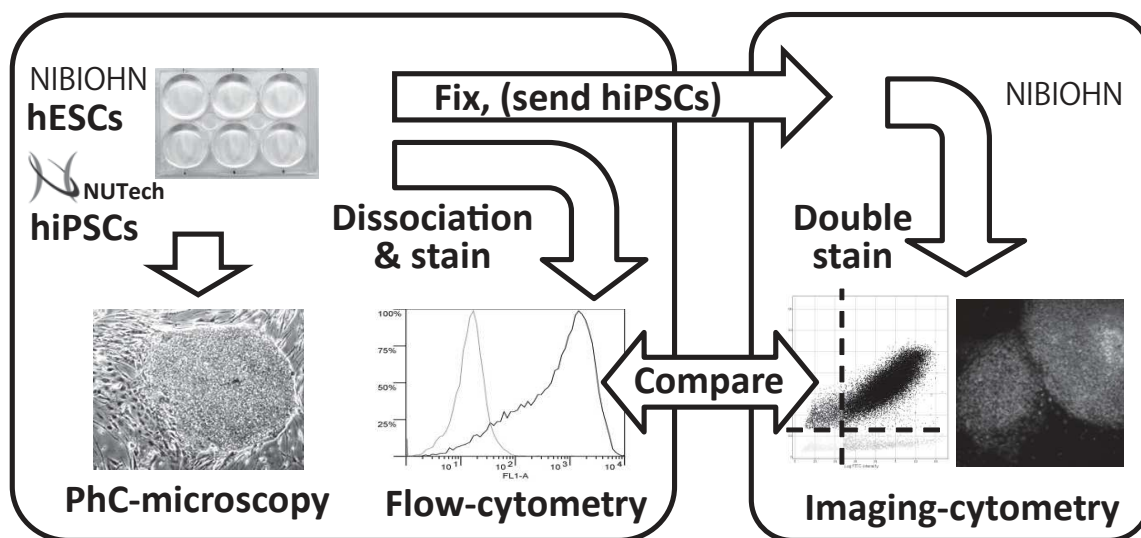

Fig. S1

Supplement: Supplementary file 2 — (PDF 72 kb) [file 11626_2016_84_MOESM2_ESM.pdf]

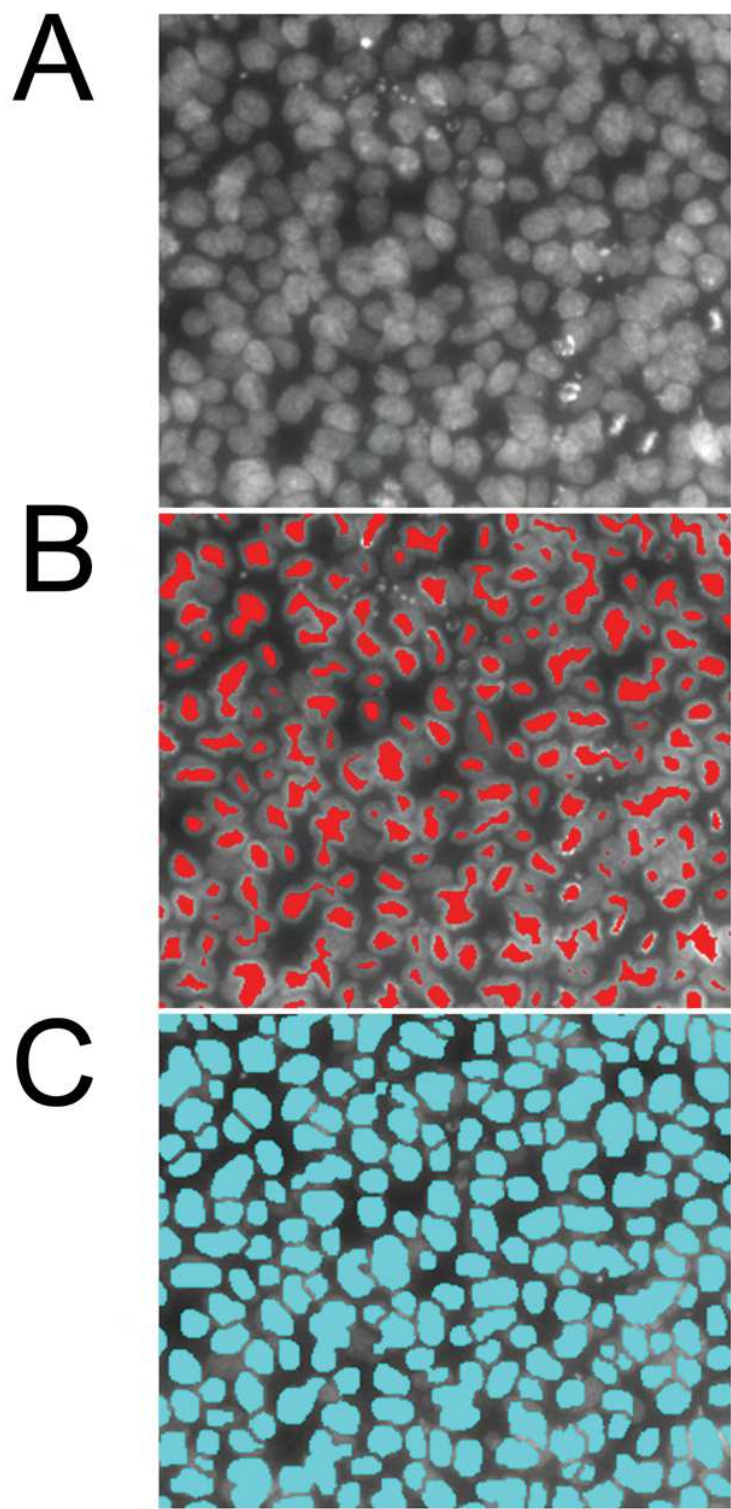

Fig. S2

Supplement: Supplementary file 3 — (PDF 112 kb) [file 11626_2016_84_MOESM3_ESM.pdf]

A

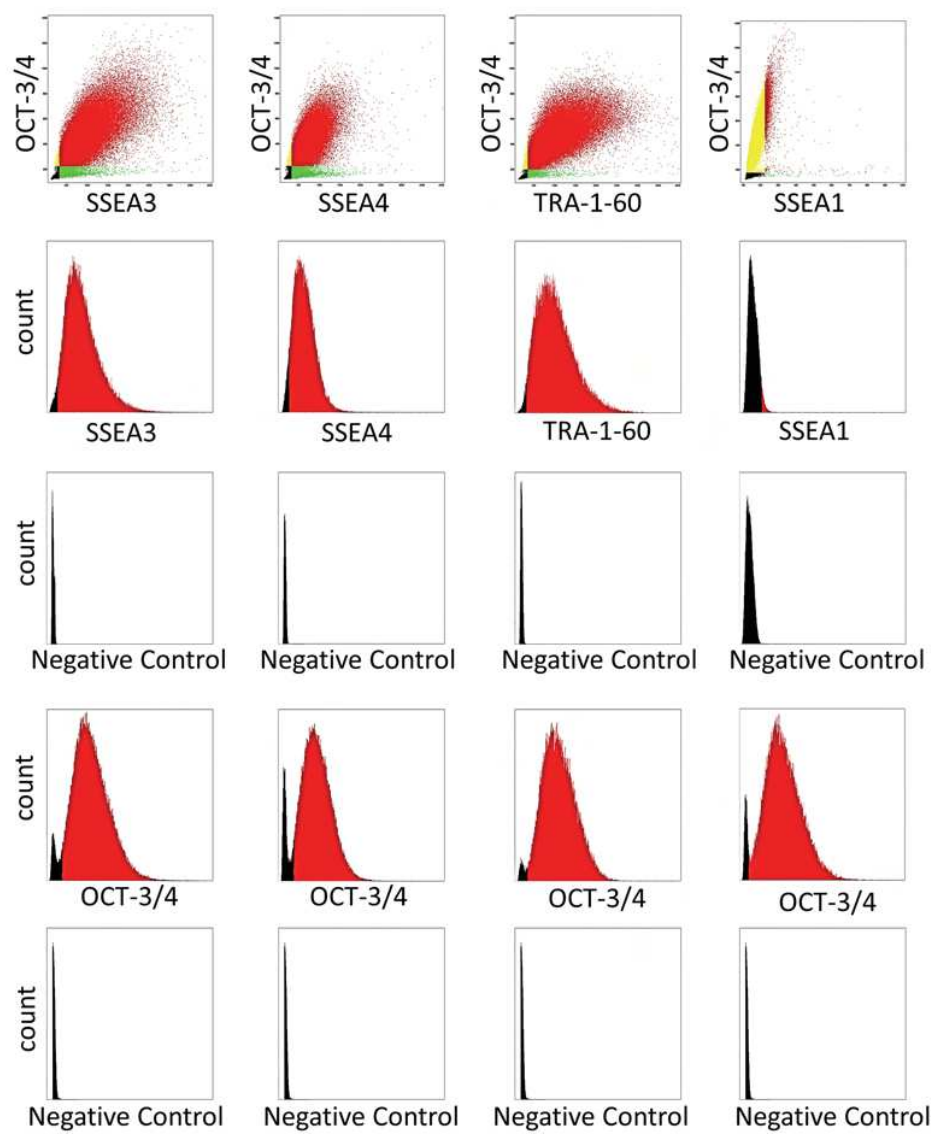

253G1

Fig. S3

B

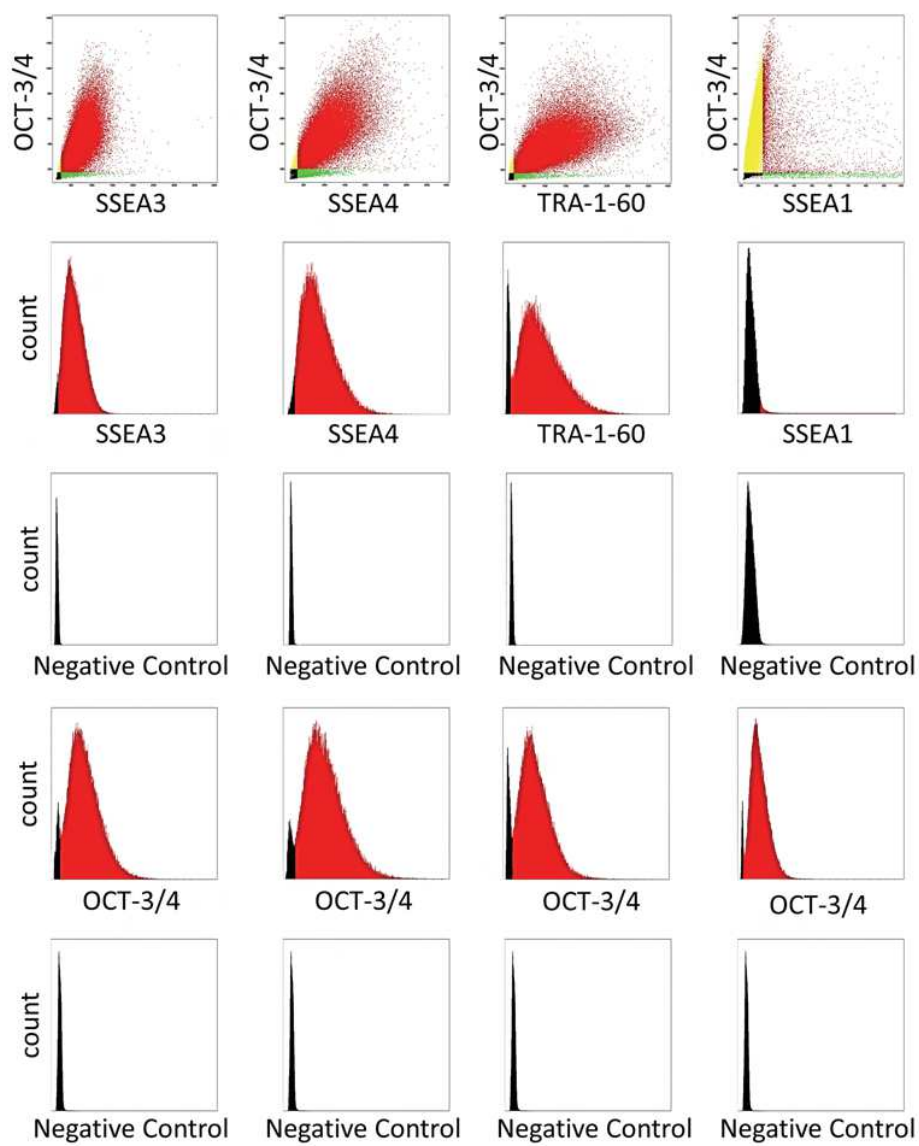

Tic

Fig. S3

C

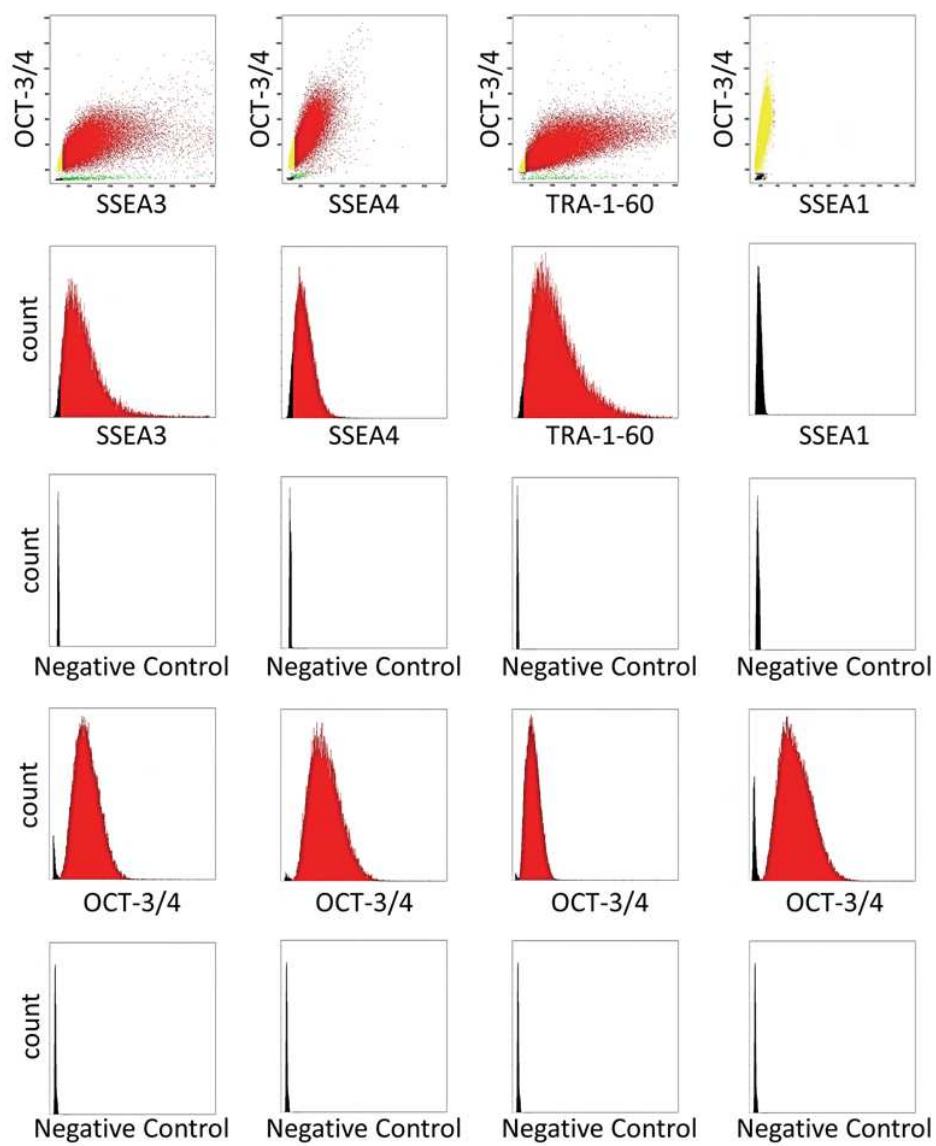

H9

Fig. S3

Supplement: Supplementary file 4 — (PDF 269 kb) [file 11626_2016_84_MOESM4_ESM.pdf]

Hoechst33342

SSEA1

OCT-3/4

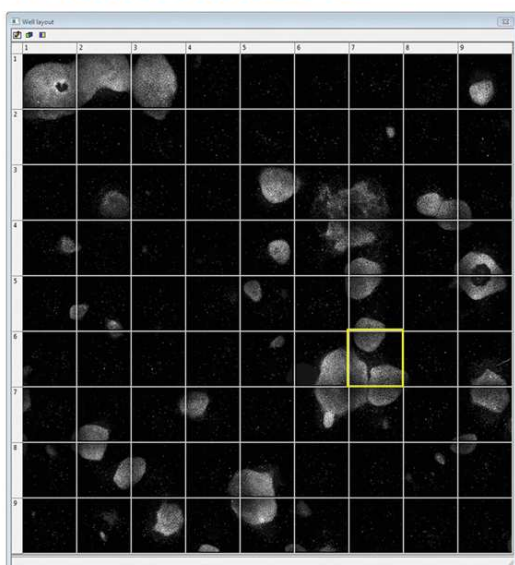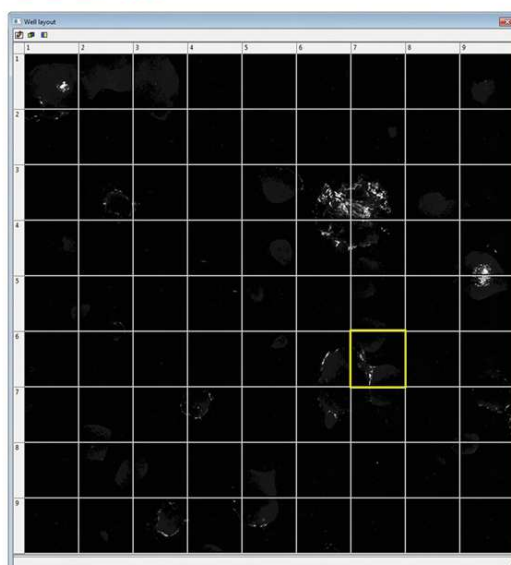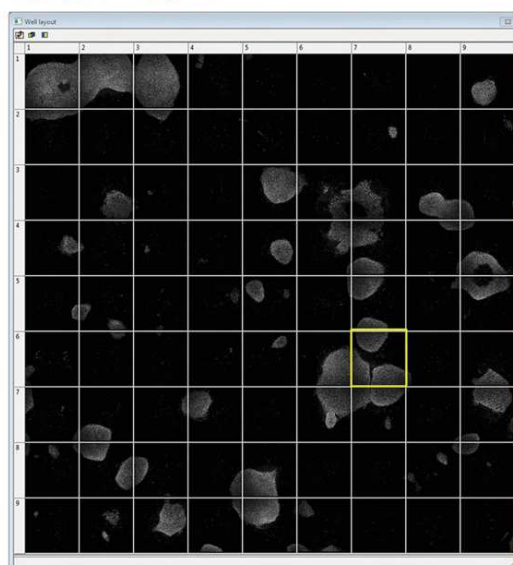

Fig. S4 (A)

Hoechst33342

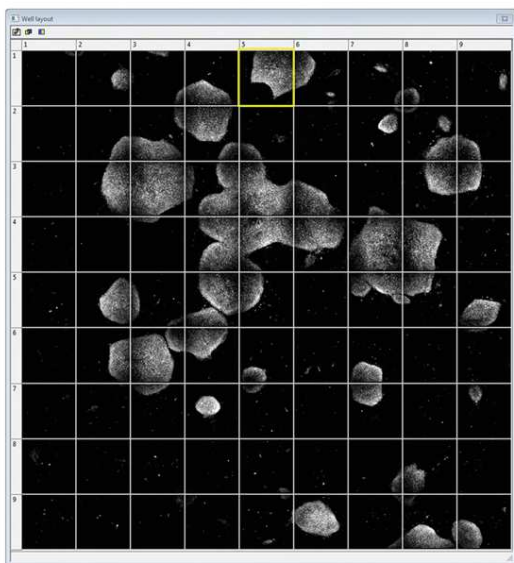

SSEA3

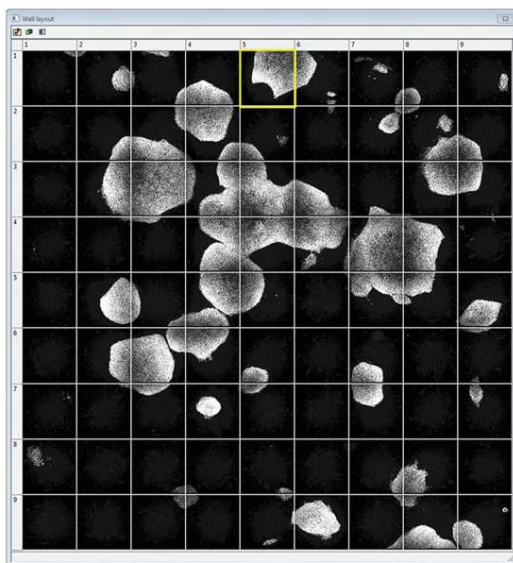

OCT-3/4

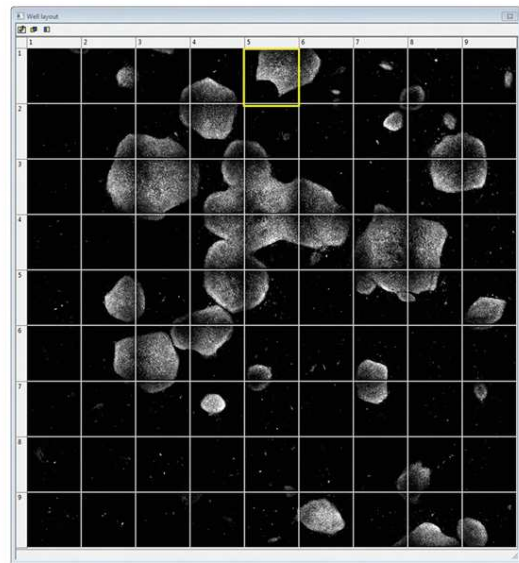

Fig. S4 (B)

Supplement: Supplementary file 5 — (PDF 291 kb) [file 11626_2016_84_MOESM5_ESM.pdf]
