# Supplementary material for: Imaging-cytometry revealed spatial heterogeneities of marker expression in undifferentiated human pluripotent stem cells
Source: In Vitro Cell Dev Biol Anim. 2016 Aug 29;53(1):83–91. doi: 10.1007/s11626-016-0084-3 (PMC5258813; doi:10.1007/s11626-016-0084-3)
Supplement: Supplementary file 6 — (PDF 41 kb) [file 11626_2016_84_MOESM6_ESM.pdf]

Supplementary Table S1. Information of hPSC lines used in this study

Cell Line:	201B7	253G1	Tic	H9
Distributor	RIKEN BRC, Japan	RIKEN BRC, Japan	JCRB Cell Bank, Japan	WISC Bank, USA
Distribution catalogue number	HPS0063	HPS0002	JCRB 1331	(WA09)H9-DL-7
Establisher	Yamanaka, S.<br>CiRA, Kyoto University [2]	Yamanaka, S.<br>CiRA, Kyoto University[12]	Umezawa, A.<br>National Center for Child Health and Development, Tokyo[13]	Thomson, J.<br>Wisconsin Univerisity[1]
Origin tissue	Human dermal skin fibroblast	Human dermal skin fibroblast	Human fetus lung fibroblast	Human embryo
Passage number of cells distributed [a]	p27	p23	p21+15	p26
Institution where cells were cultured in this study	NUTech	NUTech	NUTech	NIBIOHN
Passage number of cells analysed in this study [a]	p27+8+9<br>p27+8+10<br>p27+8+13	p23+4+5+13<br>p23+4+5+14<br>p23+4+5+15	p21+15+4+10<br>p21+15+4+11<br>p21+15+4+12<br>p21+15+4+13	p26+3+13+4<br>p26+3+13+6<br>p26+3+13+7
Culture period after obtaining cells from a cell bank	< 6 months	< 6 months	< 6 months	> 6 months
Cell authentication (STR analysis) after distribution	N/A	N/A	N/A	p26+3+13+3<br>JCRB Cell Bank [b]

a: “P” numbers designate passages number and ‘+’ designate freezing and defrosting.

b: STR DNA profile analysis was submitted to Editor.
