# Supplementary material for: Imaging-cytometry revealed spatial heterogeneities of marker expression in undifferentiated human pluripotent stem cells
Source: In Vitro Cell Dev Biol Anim. 2016 Aug 29;53(1):83–91. doi: 10.1007/s11626-016-0084-3 (PMC5258813; doi:10.1007/s11626-016-0084-3)
Supplement: Supplementary file 7 — (PDF 51 kb) [file 11626_2016_84_MOESM7_ESM.pdf]

Supplementary Table S2. Antibody information

Markers	Primary antibody	Secondary antibody
TRA 1-60	anti-human TRA-1-60 (TRA-1-60)	goat anti-mouse IgM (μ chain)
	mouse monoclonal IgM	Alexa Fluor 488
	sc-21705, SantaCruz [1]	A21042, Life Technology [2]
	Dilution 1/100	Dilution 1/500
OCT-3/4	anti-human OCT-3/4OCT-3/4 (H-134)	goat anti-rabbit IgG(H+L)
	rabbit polyclonal IgG	Alexa Fluor 546
	sc-9081, SantaCruz [1]	A11035, Life Technology [2]
	Dilution 1/500	Dilution 1/2000
SSEA4	anti-human SSEA-4(813-70)	goat anti-mouse IgG3 (γ3)
	mouse monoclonal IgG3	Alexa Fluor 488
	sc-21704, SantaCruz [1]	A21151, Life Technology [2]
	Dilution 1/100	Dilution 1/500
SSEA-3	Anti-human/mouse SSEA-3(MC-631)	goat anti-rat IgM (μ chain)
	rat monoclonal IgM	Alexa Fluor 488
	MAB1434, R&D SYSTEMS [3]	A21212, Life Technology [2]
	Dilution 1/100	Dilution 1/500
SSEA1	Anti-human SSEA-1(480)	goat anti-mouse IgM (μ chain)
	mouse monoclonal IgM	Alexa Fluor 488
	sc-21702, SantaCruz [1]	A21042, Life Technology [2]
	Dilution 1/100	Dilution 1/500
MEF	Anti-Feeder antibodies	
	Mouse conjugated to PE	
	130-096-094, Miltenyi Biotec [4]	
	Dilution 1/20	

[1] SantaCruz Biotechnology, CA

[2] Life Technology, CA

[3] R&D SYSTEMS, MN

[4] Miltenyi Biotec, Bergisch Gladbach, Germany
