# Supplementary material for: Imaging-cytometry revealed spatial heterogeneities of marker expression in undifferentiated human pluripotent stem cells
Source: In Vitro Cell Dev Biol Anim. 2016 Aug 29;53(1):83–91. doi: 10.1007/s11626-016-0084-3 (PMC5258813; doi:10.1007/s11626-016-0084-3)
Supplement: Supplementary file 8 — (PDF 58 kb) [file 11626_2016_84_MOESM8_ESM.pdf]

Supplementary Table S3. Steps in nuclear segmentation

Step	Process	Parameters
Nuc_center		
01	Segmentation (Object segmentation)	Kernel Size: 7, Sensitivity: 77
02	Fill Holes	
03	Erosion (Binary)	Kennel Size: 5
Nuc		
04	Segmentation (Object segmentation)	Kernel Size: 7, Sensitivity: 77
05	Fill Holes	
06	Erosion (Binary)	Kernel Size: 3
07	Dilation (Binary)	Kernel Size: 6
08	Watershed clump breaking (with seed)	Seed image: Nuc_center
09	Sieve (Binary)	Exclude targets with an area less than 20 μm^2 or larger than 500 μm^2

Both Nuc_center and Nuc operation use a variation of the “top-hat” approach for segmentation and banalization (**Step 01 and 04**), followed by a hole filling procedure (**Step 02 and 05**). Then the Nuc_center procedure uses an Erosion procedure to segment the center of nuclei (**Step 03**). On the other hand, the Nuc procedure use an opening procedure (Erosion and Dilation) to smooth the contours of the segmented nuclei (**Step 06 and 07**), followed by a Watershed procedure to separate objects from adjacent nuclei by using Nuc_center information as a “seed” (**Step 08**), and then a Sieve procedures to exclude small debris and large cell clumps (**Step 09**). Fluorescent intensity of Hoechst33342 (Nuclei, DAPI channel [Ch1]), Alexa Fluor 488 (anti-SSEA3, -SSEA4, -TRA-1-60 and -SSEA1 antibodies, FITC channel [Ch2]), PE (anti-Feeder antibody, Cy3 channel [Ch3]), and Alexa Fluor 647 (anti-OCT 3/4 antibody, Cy5 channel [Ch4]) were measured at the Nuc area.
