# Supplementary material for: Imaging-cytometry revealed spatial heterogeneities of marker expression in undifferentiated human pluripotent stem cells
Source: In Vitro Cell Dev Biol Anim. 2016 Aug 29;53(1):83–91. doi: 10.1007/s11626-016-0084-3 (PMC5258813; doi:10.1007/s11626-016-0084-3)
Supplement: Supplementary file 9 — (PDF 36 kb) [file 11626_2016_84_MOESM9_ESM.pdf]

Supplementary Table S4: Percent positive cells analyzed by imaging- and flow-cytometry

Cells	OCT-3/4		SSEA3		SSEA4		TRA-1-60		SSEA1	
Passage #[a]	Image	Flow	Image	Flow	Imag	Flow	Image	Flow	Image	Flow
hiPSC 201B7										
P27+8+9	85.3	82.0	94.0	73.0	95.0	97.0	87.0	91.0	30.2	34.0
P27+8+10	89.3	88.0	96.0	79.0	99.0	95.0	94.0	97.0	9.9	46.0
P27+8+13	94.2	85.0	94.9	53.0	95.2	92.0	95.1	90.0	19.9	24.0
mean±SD	89.6±4.5	85.0±3.0	95.0±1.0	68.3±13.6	96.4±2.3	94.7±2.5	92.0±4.4	92.7±3.8	20.0±10.2	34.7±11.0
hiPSC 253G1										
P23+4+5+13	92.3	76.0	95.0	73.0	96.0	92.0	100.0	83.0	34.6	22.0
P23+4+5+14	90.8	71.0	99.0	47.0	95.0	92.0	100.0	77.0	22.1	22.0
P23+4+5+15	84.5	92.0	92.0	36.0	99.0	91.0	89.0	81.0	21.2	19.0
mean±SD	89.2±4.1	79.7±11.0	95.3±3.5	52.0±19.0	96.7±2.1	91.7±0.6	96.3±6.4	80.3±3.1	26.0±7.5	21.0±1.7
hiPSC Tic										
P21+15+4+10	84.8	50.0	86.0	76.0	93.0	91.0	84.0	95.0	12.8	22.0
P21+15+4+11	93.0	82.0	97.0	73.0	97.0	89.0	91.0	92.0	15.7	11.0
P21+15+4+12	89.0	89.0	92.0	79.0	98.0	97.0	94.0	96.0	3.0	30.0
P21+15+4+13	94.0	85.0	94.0	80.0	99.0	97.0	97.0	90.0	10.5	22.0
mean±SD	90.2±4.2	76.5±17.9	92.3±4.6	77.0±3.2	96.8±2.6	93.5±4.1	91.5±5.6	93.3±2.8	10.5±5.4	21.3 7.8
hESC H9										
P26+3+13+6	97.8	99.7	89.0	89.4	98.0	98.5	93.0	99.5	0.0	1.6
P26+3+13+7	96.5	98.8	83.0	81.5	99.0	94.1	95.0	92.6	5.3	12.7
P26+3+13+4	99.3	99.7	98.0	94.0	93.0	94.0	99.0	98.6	1.2	5.2
mean±SD	97.8±1.4	99.4±0.5	90.0±7.5	88.3±6.3	96.7±3.2	95.5±2.6	95.7±3.1	96.9±3.8	2.2±2.8	6.5±5.7

a: “P” numbers designate passages number and ‘+’ designate freezing and defrosting.
